# In Preclinical Epilepsy, GLUT1 and GFAP Dysregulation in Cells Surrounding the Third Ventricle, Including Tanycytes, Is Differentially Restored with Ketogenic Diet Treatment

**DOI:** 10.3390/nu17111824

**Published:** 2025-05-28

**Authors:** Parisa Rafiei, Huda S. Mian, Shruthi H. Iyer, Samantha B. Draves, Stephanie A. Matthews, Daniel E. Rendon, Emma J. Neesen, Madeline Dunlay, McKenna Revis, Adrianna L. Glisan, Timothy A. Simeone, Kristina A. Simeone

**Affiliations:** Department of Pharmacology & Neuroscience, Creighton University School of Medicine, Omaha, NE 68178, USA; parisarafiei@creighton.edu (P.R.); hudasaadia@gmail.com (H.S.M.); shruthiiyer@creighton.edu (S.H.I.); stephaniematthews@creighton.edu (S.A.M.); samanthadraves@creighton.edu (S.B.D.); dannyrendon@creighton.edu (D.E.R.); emmaneesen@creighton.edu (E.J.N.); madeline.dunlay@ucsf.edu (M.D.); mckenna.revis@doane.edu (M.R.); adriannaglisan@creighton.edu (A.L.G.); timothysimeone@creighton.edu (T.A.S.)

**Keywords:** tanycyte, hypothalamus, ketogenic diet, metabolic therapy, epilepsy, seizure, *Kcna1*-null mouse, Kv1.1 knockout mouse, GFAP, GLUT1, DCX

## Abstract

**Background/Objectives:** Hyperexcitable neuronal activity associated with seizures may disrupt brain homeostasis resulting in abnormal glucose and nutrient management and metabolism. Specialized ependymal cells known as tanycytes line the third ventricle wall bridging communication between the brain, CSF, and blood. Despite their positional importance, whether tanycytes are impacted by epilepsy is unknown. Here, known protein markers of tanycytes were assessed in the *Kcna1*-null mouse model of genetic epilepsy with spontaneous recurrent seizures (SRS mice). Further, whether an anti-seizure metabolic ketogenic diet (KD), previously proven effective in SRS mice, restored protein levels was determined. **Methods**: Known tanycyte proteins, including glucose transporter 1 (GLUT1), glial fibrillary acidic protein (GFAP), and doublecortin (DCX, to determine potential neurogenic differences) were examined throughout the anterior–posterior axis of the third ventricle using immunofluorescent histochemistry. **Results**: Decreased GLUT1 immunoreactivity and elevated GFAP levels were found in the SRS cohorts. The number of neurogenic DCX-expressing cells did not differ. Two weeks of KD treatment reduced GFAP to WT levels. GLUT1 remained low in KD-treated SRS mice. **Conclusions**: These data suggest that the expression of proteins important for the structure and function of tanycytes is altered in preclinical epilepsy and is differentially restored with KD treatment. Whether tanycytes actively participate in the pathophysiology of epilepsy or associated comorbidities is an intriguing possibility given their integral role in brain homeostasis.

## 1. Introduction

Tanycytes are specialized bipolar, glial-like ependymal cells located on the wall of the third ventricle (3V) [1]. They have diverse functions including the ability to sense and transport glucose, nutrients, neurotransmitters, and hormones [2], in addition to acting as stem cell progenitor cells [3]. As stewards of metabolic homeostasis, tanycytes exhibit long processes extending to the hypothalamic parenchyma, making them important liaisons between the blood, CSF, and local nuclei. The four identified subtypes [1] are distributed along the dorsal–ventral axis: the dorsomedial ependymal cells and α1 tanycytes, and the ventromedial α2 tanycytes, line the ventricular walls [4]; the β1 and β2 tanycytes comprise the ventral-most aspect around the infundibular recess, the area of ventromedial arcuate nucleus, and the median eminence of the hypothalamus [4].

Epilepsy is one of the most common neurological disorders, affecting approximately 1–2% of people worldwide, and is characterized by spontaneous recurrent seizures (SRSs) [5]. Deficiencies in the transport and metabolism of glucose and nutrients such as vitamins B6, C, D, and E are associated with acquired and genetic epilepsies [6,7]. Despite the clear impact of deficient glucose and nutrient management on central nervous system excitability and the importance of tanycytes in transport and metabolism, a role for tanycytes in epilepsy is unknown. Tanycytes express structural and functional proteins that distinguish them from other local cell types, including GFAP, GLUT1, DCX, Vimentin, Nestin, and SOX2 [1,2,3,4,8,9,10,11,12,13,14]. It is commonly reported that seizure-associated neuropathology in other regions of the brain involves significant changes in the expression of GLUT1, GFAP, and DCX [15,16,17,18,19]. Using *Kcna1*-null mice, a model of severe, genetic epilepsy with SRS [20,21,22,23], we report differential changes in these proteins around the 3V.

Despite the development of first-, second-, and third-class medications for epilepsy, approximately 30–40% of the patients do not respond to anti-seizure medications [24]. In such instances, alternative metabolic therapies such as the ketogenic diet (KD), a high-fat, low-carbohydrate, and adequate-protein therapy, have proven highly effective in pharmacoresistant epilepsies [7]. Previous reports found that KD therapy reduces seizures, is neuroprotective, and prolongs life in *Kcna1*-null mice [20,21,22,23]. Thus, whether KD treatment reversed SRS changes in tanycyte protein expression is reported.

## 2. Materials and Methods

### 2.1. Animals

Congenic *Kcna1* heterozygous mice (C3HeB/FJ background strain) were bred in an animal facility room at Creighton University to generate *Kcna1*-null and wildtype (WT) littermates. All *Kcna1*-null mice begin experiencing SRS during the third postnatal week [15,20]. Male and female *Kcna1*-null mice were compared to WT littermate controls. To avoid confounding stress variables, mice were kept in a quiet, temperature (25 °C)- and humidity (50–60%)-controlled pathogen-free room on a 12 h light/dark cycle and provided food and water ad libitum. Genotypic determination of the mice was performed via tail clips by Transnetyx, Inc. (Cordova, TN, USA). Animal care, monitoring, and procedures were in accordance with National Institutes of Health Guidelines, the United States Public Health Service’s Policy on Humane Care and Use of Laboratory Animals, and were approved by the Institutional Animal Care and Use Committee at Creighton University School of Medicine. All experimental designs were in accordance with the ARRIVE 2.0 guidelines.

### 2.2. Ketogenic Diet

Beginning on P30–32, SRS mice were randomly assigned to be administered either a ketogenic diet (KD; 6.3:1, fat to carbohydrates plus proteins; Bio-Serv F3666, Frenchtown, NJ, USA) or a control standard diet (SD; 2018S Teklad Global 18% Protein Rodent Diets, Inotiv, Madison, WI, USA) for two weeks, as previously described [20,21,23].

### 2.3. Tissue Preparation

On P46–47 mice, were deeply anesthetized with isoflurane and sacrificed by transcardial perfusion with 9% NaCl followed by 4% paraformaldehyde in 0.1 M phosphate buffer (PB). Perfusions occurred at the same time of day to avoid confounding circadian variables. The brains were extracted and post-fixed overnight at 4 °C, sequentially cryoprotected with 15% and then 30% sucrose in 0.01 M PB saline (PBS), and frozen in methyl butane on dry ice. Free-floating coronal sections (30 μm) from Bregma coordinates −0.3 mm through −2.1 mm were collected (Leica CM3050S, Wetzlar, Germany) in 0.01 M PBS and stored at 4 °C. Sections were used within 18 days.

### 2.4. Immunohistochemistry

Tanycytes express glucose transporter 1 (GLUT1), glial fibrillary acidic protein (GFAP), and doublecortin (DCX). Protein levels were examined throughout the anterior–posterior aspects of the 3V in sections selected by unbiased systematic random sampling [23] from Bregma coordinates −0.82 mm through −2.06 mm. Protocols were optimized to maximize the signal to noise ratio for each protein. Overall, sections were washed with 0.01 M PBS, subjected to blocking and epitope retrieval, incubated with primary antibodies overnight (unless otherwise noted) and then with the secondary antibody, and washed with PBS. Sections were mounted onto subbed slides and coverslipped with Fluoromount G mounting media (SouthernBiotech, Birmingham, AL, USA). Specific details for each antibody are as follows. *GLUT1:* fixed in 4% PFA for 5 min; exposed to PBS with 0.3% Triton X-100 (PBST) for 30 min, 10% Normal Goat Serum (NGS) in 0.2% PBST for 1 h, rabbit anti-GLUT1 (1:500; ab652; Abcam, Cambridge, UK) in 1% NGS/0.2% PBST overnight at 4 °C, and then AF488 conjugated goat anti-rabbit IgG (1:500; A11008, Invitrogen, Waltham, MA, USA) for 3 h. *GFAP*: exposed to 20% methanol in PBS for 15 min, 10% NGS in 0.2% PBST for 30 min and AF 488 conjugated mouse anti-GFAP antibody (1:500, EMD Millipore Corp. (Burlington, MA, USA): 3991469) in 1% NGS/0.2% PBST and overnight at 4 °C. *DCX*: sections were exposed to 3% H_2_O_2_ in PBS for 30 min, 3% NGS in 0.3% PBST for 1 h, rabbit anti-DCX antibody (1:500; Cell Signaling Technology (Danvers, MA, USA): 4604S) in 0.3% PBST overnight at 4 °C, and AF 594 goat anti-rabbit (Invitrogen: A11012) in 0.2% PBST for 2 h. Negative control sections were not exposed to either the primary or the secondary antibody and positive controls included hippocampal sections, constituting a region that typically expresses GFAP, DCX, and GLUT1.

### 2.5. Study Design

The WT control cohort was compared with SRS mice treated with either SD or KD (subject *n* = 6 mice per cohort). From each cohort, anatomically matched sections throughout the anterior, middle, and posterior aspects of the 3V were processed simultaneously for each protein. Based on our histological studies [23,25], section *n* = 1–3 sections from the anterior, middle, and posterior aspects = 3–9 sections processed/subject.

### 2.6. Inclusion/Exclusion Criteria

Inclusion criteria included well-perfused brains, anatomically matched sections that were processed within 18 days. Exclusion criteria included brains that were not well-perfused or sections that had been free-floating for more than 18 days; were processed during a failed experiment, as determined by positive and negative control staining; following processing, or had folds, tears, bubbles, or dust in the region of interest. Following exclusion criteria, data from 26 mice were used in the study.

### 2.7. Image Acquisition and Analyses

Images were acquired with the Nikon Eclipse Ci-L microscope (Tokyo, Japan) located at the histology core at Creighton University. Background was subtracted from the image and haze reduction was applied. GFAP images were analyzed using ImageJ 1.53m software (Rasband, W.S., ImageJ, U. S. National Institutes of Health, Bethesda, MD, USA, https://imagej.net/ij/, 1997–2018, accessed on 15 September 2022). Different methods of analysis were used to properly quantify the unique expression pattern of each protein. GFAP immunoreactivity (IR) was apparent in the cell bodies surrounding the 3V and their respective proximal processes. In some sections, the walls of the 3V were superimposed (due to mounting SOP dictating minimal adjustments), and thus, to control for the heightened signal, somatic fluorescence was not quantified. For GFAP, the signal was converted into a binary signal and the total sum of the pixels comprising the skeletonized signal was analyzed. GLUT1-IR was notable in the cell bodies surrounding the 3V and in the rootlets at the base of their processes that extended into the parenchyma. To control for ventricular wall superimposition, the number of GLUT1-positive rootlets were manually quantified (GLUT1-IR in hypothalamic capillaries were excluded). Tanycytes are neurogenic and DCX-IR was apparent in neural precursors in hypothalamic tissue lateral to the 3V (as well as in the hippocampus, which served as a positive control). The number of DCX-IR cells was manually quantified. To ensure rigor and reproducibility, regions of interest in all images were quantified at least twice by at least two blinded investigators (training of image analysis techniques involved demonstration of within (self) and between (investigators) variability of <5%). Discrepancies were discussed and unanimously decided by at least three blinded investigators.

### 2.8. Statistical Analysis

Data are presented as means ± SEMs. Statistical analysis among groups was determined with GraphPad Prism10 software (GraphPad Software Inc., La Jolla, CA, USA) using ANOVA followed by an appropriate post hoc test to account for unequal variance if necessary (see figure legends for statistical tests). 

## 3. Results

### 3.1. The Number of Glut1-IR Rootlets Was Reduced in SRS Mice

**Figure 1:**
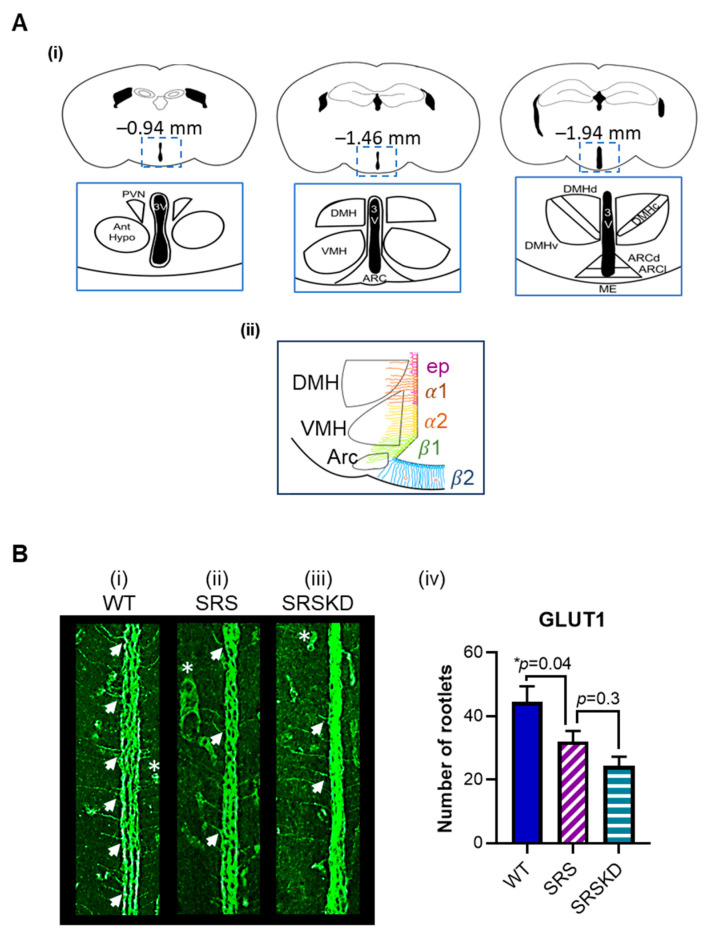
Schematic of the 3V and representative images of GLUT1-IR from WT, SRS, and SRS KD cohorts. (**A**) (i) A cartoon depicting representative anatomical landmarks at anterior, middle, and posterior atlas coordinates and (ii) the distribution of ependymal cells and α1, α2, β1, and β2 tanycytic subtypes along the 3V at the level of the median eminence (capillaries indicated by red circles) (drawing by author H.S.M). (**B**) (i–iii) Digital magnification of representative images of 3V GLUT1-IR in WT, SRS, and SRSKD cohorts. GLUT1-positive rootlets are indicated with arrows. GLUT1-positive capillaries are indicated with asterisks (not quantified). Note: The higher GLUT1-IR ventricular signal in SRSKD was due to ventricular wall superimposition (somatic IR was not quantified). (iv) Quantification of GLUT1 rootlets in WT, SRS, and SRSKD groups using ANOVA (F (2, 78) = 6.8, *p* = 0.002) with Dunnett’s multiple-comparison test, * p < 0.05; *n* = 3–9 sections per mouse, 6 mice per cohort. Abbreviations: 3V, third ventricle; Ant Hypo, anterior hypothalamus; ARC, arcuate nucleus; ARCd, dorsal arcuate nucleus; ARCl, lateral arcuate nucleus; DMH, dorsomedial hypothalamic nucleus; DMHc, dorsomedial hypothalamic nucleus central; DMHd, dorsomedial hypothalamic nucleus dorsal; DMHv, dorsomedial hypothalamic nucleus ventral; ME, median eminence; PVN, paraventricular nucleus; VMH, ventromedial hypothalamic nucleus.

Representative anatomical landmarks at anterior, middle, and posterior Bregma coordinates and the approximate distribution of ependymal cells and α1, α2, β1, and β2 tanycytic subtypes surrounding the 3V are depicted in Figure 1A. The GLUT 1 protein is expressed throughout the dorsal–ventral aspects of the 3V wall and facilitates transportation of glucose and the oxidized form of vitamin C, dehydroascorbic acid, into the brain [8]. As the functional implications of the subtypes are not fully understood, GLUT1 immunoreactivity (IR) within the dorsal aspect and ventral aspect of the 3V walls was assessed. GLUT1-IR was apparent in the cell bodies, rootlets, and processes extending into hypothalamic parenchyma in middle and posterior aspects of the 3V (Bregma coordinates −1.05 to −1.50 mm). The high somatic protein expression appeared similar in all cohorts and was not quantified to avoid confounding and artificially high fluorescent signals from sections with left and right 3V wall superimposition. Upon further examination, the numbers of GLUT1-positive rootlets extending into the parenchyma were visibly different among cohorts at Bregma-matched sections and were manually quantified. SRS mice had fewer GLUT1-IR rootlets when compared with WT controls (WT 45 ± 5 vs. SRS 32 ± 3 rootlets, *p* < 0.05) (Figure 1B(i,ii,iv)). Differences between dorsal and ventral aspects among cohorts were not noted.

### 3.2. GFAP Was Increased in SRS Mice

**Figure 2:**
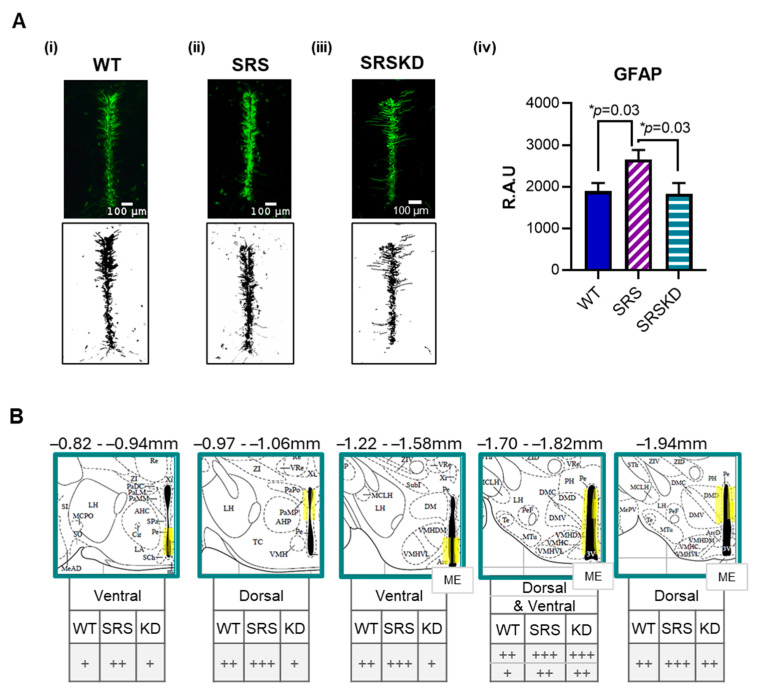
GFAP-IR from WT, SRS, and SRS KD cohorts. (**A**) (i–iii) Representative images of 3V GFAP-IR from WT, SRS, and SRSKD cohorts. The respective digital skeletonization image is below. (iv) Quantification of skeletonized GFAP in relative arbitrary units (RAU). To account for unequal distribution of variance, Brown–Forsythe ANOVA (F (2, 67) = 4.3, *p* = 0.02) with Dunnett’s multiple-comparison test was used, * *p* < 0.05, *n* = 3–9 sections per mouse, 6 mice per cohort. (**B**) At different Bregma coordinates (Paxinos and Watson Atlas), semi-quantitative assessment indicated nuanced increases in GFAP either at dorsal and/or ventral aspects of 3V wall (highlighted in yellow) in SRS mice, which were reversed in KD cohorts (+ minimal, ++ moderate, +++ pronounced). The exception was the increase at −1.70–−1.82 mm in both dorsal and ventral aspects, which did not appear to be rescued in SRSKD mice.

Glial Fibrillary Acidic Protein (GFAP) is a type III intermediate filament protein that is mainly expressed by astrocytes and tanycytes [9,10,11] (primarily by α1 and α2 tanycytes [12], but also in β-tanycytes [3]). Following the skeletonization of GFAP fluorescence, the binary signal was quantified (in relative arbitrary units). GFAP was significantly increased in SRS mice when compared to WT controls (WT 1892 ± 199 vs. SRS 2658 ± 224 RAU) throughout the anterior, middle, and posterior aspects of the 3V (Figure 2A). The signals from the dorsal and ventral aspects of the 3V wall were stratified. Semi-quantitative assessment indicated that increased GFAP was more pronounced at the ventral aspects at Bregma −0.82 through −0.94 and Bregma −1.22 through −1.58 mm (Figure 2B). In contrast, at the dorsal aspect, GFAP was more prominent at Bregma −0.97 through −1.06 and −1.94 mm. Interestingly, both the ventral and dorsal walls had elevated GFAP levels at Bregma −1.7 through −1.82 mm (Figure 2B).

### 3.3. KD Treatment Did Not Influence GLUT1-IR but Did Restore GFAP Levels in SRS Mice

Previous studies had demonstrated that a two-week treatment of KD reduced seizures and was neuroprotective in SRS mice [20,22,23]. Contrary to the multitude of beneficial effects reported for KD, the number of GLUT1-IR rootlets did not differ between SRS and SRSKD cohorts (SRS 32 ± 3 vs. SRSKD 24 ± 3 rootlets, *p* = 0.3). However, KD treatment did rescue and reduce GFAP levels around the 3V in SRSKD mice when compared with SRS alone (SRS 2658 ± 225 vs. SRSKD 1832 ± 256 RAU, *p* = 0.03) to levels that resembled those of WT controls.

### 3.4. The Number of DCX-IR Cells Did Not Differ Between WT and SRS Groups

GFAP-positive dorsal α-tanycytes and β-tanycytes [26] are reportedly neurogenic. Doublecortin (DCX) is a microtubule-associated protein that is expressed by migrating neural precursor cells during development and adult neurogenesis [27,28]. DCX-IR is observed in the dentate gyrus of the hippocampus and tanycytes surrounding the 3V [13]. The number of DCX-IR cells within the 3V ventricular wall and in the surrounding parenchyma (~500 µm lateral to 3V) to account for migrating cells was quantified, and we found no significant differences between WT and SRS cohorts (WT 2.7 ± 0.7 vs. SRS 2.2 ± 0.5 cells, *p* > 0.05). Since groups did not differ, the effect of KD treatment was not assessed. Interestingly however, when data were collapsed across genotypes, there were more DCX-IR cells in the middle + posterior hypothalamus when compared to the anterior sections (anterior 1.4 ± 0.5 vs. mid + post 5.1 ± 1.0 cells, *p* < 0.001, unpaired *t* test).

## 4. Discussion

Due to their distinct morphology and function, tanycytes are considered metabolic integrators that are crucial for regulating neuronal functions and maintaining energy balance [1,2,3,4]. In addition, tanycytes interact with the Blood–Brain Barrier (BBB) in multiple ways including monitoring neurohormone release from the hypothalamus and transporting hormones such as insulin [2]. As seizures are metabolically demanding, and metabolic dysregulation is associated with epilepsy, the probability of a potential role for tanycytes is high; however, it has yet to be explored. Here, a straightforward immunohistochemical study was conducted to determine whether gross differences in important markers of tanycytes were detectable between control and epileptic animals. Tanycytes express proteins differentiating them from other cell types surrounding the 3V. The double-labeling of useful markers includes, but is not limited to, GFAP, GLUT1, DCX, Vimentin, Nestin, and SOX2 [1,2,3,4,8,9,10,11,12,13,14]. It is commonly reported that seizure-associated neuropathology in other regions of the brain involves significant changes in the expression of GLUT1, GFAP, and DCX [15,16,17,18,19]; therefore, these three markers were examined. Data herein indicated, for the first time, that around the 3V in SRS mice, there was (i) a significant reduction in GLUT1-positive rootlets that extend processes into the hypothalamic parenchyma, and (ii) an increase in GFAP-IR, but (iii) no difference in DCX-positive cells. Furthermore, (iv) treatment with an anti-seizure metabolic KD therapy restored GFAP levels but had no effect on GLUT1.

GLUT1 is expressed by several different cell types in the brain including tanycytes, ependymal cells, endothelial cells of the BBB, and astrocytes [14,29]. GLUT1 facilitates the transport of glucose and the oxidized form of vitamin C, dehydroascorbic acid, across the BBB [30]. Functional mutations in the GLUT1 gene (SLC2A1) are associated with human epilepsy [16]. The disruption of glucose transport results in insufficient glucose in the brain [16,31], and this leads to the hyperexcitability of neurons and seizures [17]. This was the first study to report a decrease in GLUT1 expression in preclinical epilepsy that was not caused by a GLUT1 genetic deficiency. Data herein may suggest that abnormal GLUT1 expression at the 3V may further propagate the pathophysiology that contributes to generation of seizures in SRS mice.

Common treatments for epilepsy associated with GLUT1 deficiency syndromes include metabolic therapies because they provide non-glucose sources for energy [32]. KD is highly effective at reducing seizures in people with GLUT1 deficiency and is the first-line treatment [32]. The primary energy sources of KD are fatty acids and ketone bodies. Fatty acids have been shown to increase the expression of GLUT1 in adipocytes [33] via the transcription factor PPARgamma [34], and the gene regulation effects of a KD are known to involve the activation of PPARgamma [21]. Interestingly, a two-week KD treatment did not impact the number of GLUT1-IR rootlets despite previous studies demonstrating that this treatment duration sufficiently attenuated seizures and was neuroprotective in SRS mice [20,21,22,23]. This supports the notion that ketone bodies provided by the KD contribute significantly to its antiseizure efficacy by acting as a fuel source in place of glucose, thereby circumventing the GLUT1 deficiency [7]. Alternatively, the lack of an observable effect could have been due to initiating KD treatment at P30, an age when SRS occur daily and CNS damage/remodeling may have already begun. Future studies will adjust the timing and duration of KD treatment to determine whether longer treatment is necessary for additional disease-modifying gene regulation effects on GLUT1 expression.

GFAP is an intermediate filament expressed by several cell types in the brain such as astrocytes, radial glia [35], and the tanycyte-like ependymal cells of the 3V [12]. It is a component of the cytoskeleton assisting in shape and movement of processes. An increase in GFAP levels is observed in epilepsy and other neurological diseases in reactive astrocytes and astrogliosis and is detrimental to their participation in maintaining ion homeostasis [36,37]. GFAP expression was uniquely impacted in SRS mice at the ventral and/or dorsal aspects of the 3V throughout the anterior–posterior axis in a Bregma-specific manner. In contrast to reactive astrocytes and astrogliosis, the elevated GFAP levels were not associated with morphological changes in tanycytes. The metabolic treatment with KD of SRS mice did restore GFAP expression to WT levels, supporting the influence of ketosis on glial morphology [38].

It is unclear whether the reduction in GLU1 rootlets and elevated GFAP are associated with hypothalamic pathophysiology. If the changes in GLUT1 or GFAP at the 3V are pathologic, as crucial liaisons of glucose, nutrients, neurotransmitters, and hormones [2] with hypothalamic nuclei, tanycyte dysfunction may manifest as Bregma-dependent comorbidities [39]. For example, an early change in GFAP was first apparent at Bregma −0.82–−0.94 mm in the ventral aspect of the 3V wall. Dysregulation here may potentially impact central and peripheral circadian rhythms (suprachiasmatic nucleus), thermoregulation (anterior hypothalamus), or water balance (arginine vasopressin hormone release from the supraoptic nucleus). Moving towards the middle region (Bregma −0.97–−1.06 mm), impacted dorsal tanycytes may influence sympathetic autonomic function, stress responses, thyroid function, parturition, lactation, or water balance (paraventricular nucleus). Ventral dysregulation along the 3V walls noted at the level of the infundibulum (−1.22–−1.58 mm) may impact neuronal function in the arcuate nucleus (neuroendocrine regulation) or in the ventromedial nucleus (sexual behavior receptivity in females, and satiety). Elevated GFAP along the entire dorsal and ventral aspects of the 3V in the posterior hypothalamus (at Bregma −1.70–−1.82 mm) may influence gastrointestinal function, blood pressure, heart rate, thirst, appetite, body weight, and the cardiovascular response to stress (dorsomedial nucleus) or functions associated the arcuate and ventromedial nuclei (above). In addition, if the metabolic impact of tanycytes reaches beyond the medial aspects of the hypothalamus to the lateral hypothalamic nucleus, sleep, waking, and feeding behavior may be impacted as well (via orexin and melanocortin hormone neurons). Indeed, cardiorespiratory and sleep comorbidities have been reported in this model of SRS [25,40].

DCX is expressed in neurogenic cells such as those in the subventricular zone and the subgranular zone of the dentate gyrus in the hippocampus [41]. Previous preclinical and clinical studies have reported increased in neurogenesis in the hippocampus of epileptic humans and animals, that leads to ectopic granule cells in the dentate gyrus, which participates in hippocampal hyperexcitability [18,19,41,42]. Tanycytes have been demonstrated to also act as neural stem/progenitor cells capable of migrating and differentiating into both neurons and glial cells [43]. Our results indicate that while there were more DCX-IR cells in the middle–posterior aspects of the hypothalamus, there was no difference between WT and SRS mice, suggesting that seizures may not impact tanycytic neurogenesis.

In conclusion, these data suggest that the expressions of proteins important for the structure and function of tanycytes are altered in epilepsy. Whether tanycytes actively participate in the pathophysiology of epilepsy or the associated comorbidities is intriguing given their integral role in brain homeostasis. This study provided a foundation warranting future investigation into this fascinating field.

## Data Availability

The original contributions presented in this study are included in the article. Further inquiries can be directed to the corresponding author.

## Abbreviations

The following abbreviations have been used in this manuscript:

3V	Third Ventricle
DCX	Doublecortin
GFAP	Glial Fibrillary Acidic Protein
GLUT1	Glucose Transporter 1
IR	Immunoreactive
KD	Ketogenic Diet
NGS	Normal Goat Serum
PB	Phosphate Buffer
PBS	Phosphate Buffer Saline
PBST	Phosphate Buffer with Triton X-100
PFA	Paraformaldehyde
SRS	Spontaneous Recurrent Seizure
WT	Wildtype
